# SCFAs-Induced GLP-1 Secretion Links the Regulation of Gut Microbiome on Hepatic Lipogenesis in Chickens

**DOI:** 10.3389/fmicb.2019.02176

**Published:** 2019-09-26

**Authors:** Jian-Mei Zhang, Yin-Shuang Sun, Li-Qin Zhao, Tian-Tian Chen, Mei-Na Fan, Hong-Chao Jiao, Jing-Peng Zhao, Xiao-Juan Wang, Fu-Chang Li, Hai-Fang Li, Hai Lin

**Affiliations:** ^1^Shandong Provincial Key Laboratory of Animal Biotechnology and Disease Control, College of Animal Science and Veterinary Medicine, Shandong Agricultural University, Tai’an, China; ^2^Shandong Key Laboratory of Animal Microecological Agents, Biological Research Institute, Shandong Baolai-Leelai Bioengineering Co., Ltd., Tai’an, China; ^3^College of Life Sciences, Shandong Agricultural University, Tai’an, China

**Keywords:** gut microbiota, SCFAs, GLP-1, hepatic lipogenesis, MAPK, AMPK/ACC, chicken

## Abstract

The impact of gut microbiota and its metabolites on fat metabolism have been widely reported in human and animals. However, the critical mediators and the signal transductions are not well demonstrated. As ovipara, chicken represents a specific case in lipid metabolism that liver is the main site of lipid synthesis. The aim of this study is to elucidate the linkage of gut microbiota and fat synthesis in broiler chickens. The broilers were subjected to dietary treatments of combined probiotics (*Animal bifidobacterium*: 4 × 10^8^ cfu/kg; *Lactobacillus plantarum*: 2 × 10^8^ cfu/kg; *Enterococcus faecalis*: 2 × 10^8^ cfu/kg; *Clostridium butyrate*: 2 × 10^8^ cfu/kg, PB) and guar gum (1 g/kg, GG), respectively. Results showed that dietary supplementation of PB and GG changed the cecal microbiota diversity, altered short chain fatty acids (SCFAs) contents, and suppressed lipogenesis. In intestinal epithelial cells (IECs), SCFAs (acetate, propionate, and butyrate) up-regulated the expression of glucagon-like peptide-1 (GLP-1) via mitogen-activated protein kinase (MAPK) pathways, mainly via the phospho - extracellular regulated protein kinase (ERK) and phospho-p38 mitogen activated protein kinase (p38 MAPK) pathways. GLP-1 suppressed lipid accumulation in primary hepatocytes with the involvement of (AMP)-activated protein kinase/Acetyl CoA carboxylase (AMPK/ACC) signaling. In conclusion, the result suggests that SCFAs-induced GLP-1 secretion via MAPK pathway, which links the regulation of gut microbiota on hepatic lipogenesis in chickens.

## Introduction

Gut microbiota plays an important role in the metabolism of the host. The altered structure of gut microbiota can affect the energy metabolism (Bäckhed et al., 2004; Turnbaugh et al., 2006). Altered gut microbiota changes the end products of fermentation such as short chain fatty acids (SCFAs), which is suggested to be involved in the benefits of microbiota diversity on lipid metabolism (Kimura et al., 2013; Morrison and Preston, 2016), Studies demonstrated that oral administration of SCFAs such as acetate, propionate, and butyrate could reduce or reverse body weight gain and adiposity via increased energy expenditure, fat oxidation, or reduced food intake (Lin et al., 2012; Frost et al., 2014; den Besten et al., 2015).

Short chain fatty acids can serve as signaling molecules via the pathway of specific G protein-coupled receptors GPR41 (FFAR3) and GPR43 (FFAR2) (Brown et al., 2003; Covington et al., 2006). FFAR2 and FFAR3 are demonstrated to be involved in the regulation of lipid and glucose metabolism (Samuel et al., 2008; Zaibi et al., 2010). The activation of FFAR2 and FFAR3 by luminal SCFAs regulates glucagon-like peptide-1 (GLP-1) secretion (Kaji et al., 2014), but the mechanisms has not been well demonstrated. Seljeset and Siehler (2012) showed that FFARs can effectively activate ERK 1/2. Goldsmith and Dhanasekaran (2007) reported that the MAPKs signal pathways can be activated by G proteins. However, it remains unclear whether the MAPKs signal pathways were involved in the process of FFARs induced GLP-1 secretion.

Studies from human and mammals studies have shown that activation of GLP-1R could reduce the triglyceride (TG) content, decrease the levels of aspartate aminotransferase (AST) and alanine aminotransferase (ALT), and improve hepatic steatosis (Cuthbertson et al., 2012; Trevaskis et al., 2012). However, lower expression of GLP-1R limits the study of its direct effect on lipid metabolism in the liver of human and mammals.

Different from mammals, liver is the main site of lipid synthesis in chickens (Hermier, 1997). The accumulation of TG in liver easily leads to fatty liver syndrome in broilers (Mooney and Lane, 1981). Studies showed that GLP-1 could increase satiety and inhibit feed intake in chickens (Tachibana et al., 2007; Honda, 2016). It was demonstrated that intraventricular injection of GLP-1 could reduce the level of blood sugar, affect energy consumption and fat metabolism in chickens (Tachibana et al., 2007). Till now, studies of GLP-1 in poultry mainly focused on its regulation on poultry appetite. Huang et al. (2012) showed GLP-1R is also widely expressed in various tissues of broilers, including intestine and liver et al. (Huang et al., 2012). These findings suggest that besides its effect on the central nervous system to reduce appetite, GLP-1 might have a direct effect on the liver of broilers. Hence, we hypothesized that SCFAs-induced GLP-1 secretion may play a beneficial influence on hepatic lipid metabolism.

The aim of the present study is to explore the link of GLP-1 between gut SCFAs and hepatic lipid synthesis, and provide reference for intestinal microorganism and fat metabolism in mammals and humans. In the present study, broilers were fed with a diet supplemented with probiotics or a dietary fiber, guar gum. The gut microbiota diversity, cecal SCFAs contents, and GLP-1 receptor (GLP-1R) levels were measured. The direct effect of SCFAs on GLP-1 secretion was evaluated *in vitro* by using primary IECs. The regulation of GLP-1 on hepatic lipid metabolism was determined with liraglutide in cultured primary hepatocytes *in vitro*.

## Materials and Methods

### Animal Experiment

All animal experiments were approved by the Institutional Animal Care & Use Committee at College of Animal Science of Shandong Agriculture University. Animals were treated in accordance with the Chinese Act on Experiments on Animals. A total of 150 one-day-old healthy broiler (Arbor Acres) chicks were randomly divided into 3 treatment groups, with 5 replicate pens of 10 chicks, and fed with one of the three diets: high fat basal diet (HFD) as Control, feed formula of HFD was listed in Table 1. Control), HFD supplemented with combined probiotics (PB, *Animal bifidobacterium*: 4 × 10^8^ cfu/kg; *Lactobacillus plantarum*: 2 × 10^8^ cfu/kg; *Enterococcus faecalis*: 2 × 10^8^ cfu/kg; *Clostridium butyrate*: 2 × 10^8^ cfu/kg) based on our preliminary trial and previous report (Ewing and Cole, 1994), and HFD supplemented with 1g/kg guar gum (GG). Broilers were reared in an environmentally controlled room. Temperature and lighting were maintained in accordance with commercial conditions. The chickens had free access to feed and water during the whole experimental period. The composition and nutrient levels of the basal diet were listed in Table 1. All animal experiments were performed in accordance with the “Guidelines for Experimental Animals” of the Ministry of Science and Technology (Beijing, China).

**TABLE 1:** Composition and nutrient levels of the experimental diets (on an air-dry basis).

**Ingredients (%)**	**0–3 W**	**3–6 W**
Corn	52.05	54.66
Soybean meal	38.02	32.81
Soybean oil	5.79	8.5
Maifan stone powder	0.99	1.13
CaHPO_4_	2.02	1.87
NaCl	0.32	0.29
Lysine	0.1	0.12
Methionine	0.2	0.18
Choline chloride	0.26	0.2
Premix^∗^	0.25	0.25
**Calculated nutrient content**		
Crude protein	21	19
Crude fat	8	10.7
Metabolizable energy, kcal/kg	3100	3300
Calcium	0.9	0.9
Available phosphorus	0.45	0.42
Nacl	0.35	0.32
Digestible lysine	1.2	1.096
Digestible methionine	0.48	0.44
Digestible methionine and cysteine	0.791	0.728
Digestible threonine	0.828	0.744
Tryptophan	0.264	0.235
Leucine	1.547	1.432
Isoleucine	0.841	0.75
Valine	0.996	0.897

*Premix provides the following per kg of diet: VA, 8000 IU; VD 3, 3000 IU; VE, 20 IU; VK, 2 mg; VB 1, 4 mg; riboflavin, 8 mg; D-pantothenic acid, 11 mg; VB 5, 40 mg; VB 6, 4 mg; VB 12, 0.02 mg; biotin, 0.15 mg; folic acid, 1.0 mg; choline, 700 mg; Fe (as ferrous sulfate), 80 mg; Zn (as zinc sulfate), 75 mg; Mn (as manganese sulfate), 80 mg; Cu (as copper sulfate) 10 mg, I (as potassium iodide), 0.40 mg; and Se (as sodium selenite), 0.30 mg.*

At day 21 and day 42 of age, two chickens around mean body weights (21 days of age 600.0 ± 50 g; 42 days of age, 2000 ± 150 g) were selected from each pen, and 10 chickens in total were selected in each treatment. A blood sample was drawn from the wing vein using heparinized syringes. Plasma samples were obtained after centrifugation at 3,000 × *g* for 10 min at 4°C, stored at −80°C, and eight samples were randomly selected for further analysis of TG and total cholesterol (TCH) levels. Chickens were sacrificed immediately after the blood sample collection, the liver and abdominal fat pad were separated, harvested, and weighed. The liver and abdominal fat indexes were calculated as the percentage of body weight. Approximately 1 g cecum contents were collected, 1–2 g tissue samples were collected from the ileum, caecum, colorectum, liver, and abdominal adipose. All the samples were snap-frozen in liquid nitrogen, and then stored at −80°C for subsequent analysis. The tissue samples from eight chickens were randomly selected and used for the measurement of gene expression while the liver and abdominal fat tissue samples from six chickens were used for the histological observation.

### TG and TCH Content Measurements

TG and TCH contents were determined with commercial kits (GPO-PAP and CHOD-PAP, Nanjing Jiancheng Biotechnology Institute, China).

### Histological Staining

Paraffin-embedded liver and abdominal adipose tissues were sliced into 5 μm sections for hematoxylin and eosin stain (HE, Nanjing Jiancheng Bioengineering Institute, China). The histological features were observed and captured under a light microscope.

### Measurement of Cecal SCFA Concentrations

Short chain fatty acids concentrations were determined using GC–MS assay. 0.2 g cecal contents was added to 2 mL of water with phosphoric acid, vortexed and homogenized for 2 min. Then, 2 mL of ether was added to the sample, which was rested for 10 min and centrifuged at 4000 rpm for 20 min at 4°C. The ether phase was removed after centrifugation, and then the extraction was repeated. The two extracts were combined, volatilized to 2 mL, and injected into the GCMS ISQ LT (Thermo Fisher, United States) and TRACE GCMS ISQ LT (Thermo Finnigan, United States) with the following conditions: initial column temperature at 100°C, held for 5 min, increased at a rate of 5°C/min to 150°C followed by 30°C/min to 240°C, then held at 240°C for 30 min; flow rate: 1 mL/min; split ratio: 75:1; carrier gas: helium; column: TG WAX 30 m × 0.25 mm × 0.25 μm; injector: 240°C; mass spectrometry: EI source; bombardment voltage: 70 eV; single ion scanning mode: quantitative ions 60,73; ion source temperature: 200°C; cable temperature: 250°C; and quantitative analysis method: external standard curve method.

### 16S rRNA-Based Analysis of Cecal Microbiota

To analyze the composition of chicken cecal microbiota in different groups, 16S rRNA sequencing of the gut microbiome was performed in Annoroad Gene Tech. Co. (China). After the microbial DNA was extracted, the integrity of the extracted DNA was determined using electrophoresis on a 1% agarose gel. PCR amplification was performed with the TransStart FastPfu DNA Polymerase (Transgen Biotech, China). Each sample was repeated three times. PCR products from the same sample were mixed, and electrophoresis was performed with 2% agarose gel detection. The DNA extracts were used as templates to amplify the V3 hypervariable region of the 16S rDNA gene with the primers 338F (5′-ACTCCTACGGGAGGCAGC-3′) and 533R (5′-TTACCGCGCCTGCTGGCAC-3′). Three replicates were performed for each sample, each replicate consisted of 1 μL (100 ng) DNA template. The PCR products were tested with the QuantiFluor^TM^-ST blue fluorescence quantitative system (Promega, China). The MiSeq library was constructed and sequenced according to the sequencing quantity requirements of each sample. First, PE reads sequenced by MiSeq were spliced according to the overlap relationships; then, the sequence quality was qualitatively controlled and filtered to distinguish the sample from the subsequent OTU cluster analysis. Finally, a species taxonomy analysis was conducted. Based on the OTU clustering analysis results, multiple diversity index and sequencing depth analyses can be conducted using line detection. Based on taxonomic information, community structure statistics can be evaluated at various classification levels.

### Primary Culture of Chicken Intestinal Epithelial Cells

Specific pathogen-free (SPF) chicken eggs were purchased and incubated for 19 days. The chicken embryos were used for the isolation of primary duodenal IECs. The duodenal mucosa was gently extruded and transferred to Hank’s Balanced Salt Solution (HBSS) (Solarbio, China). The mucus was washed with HBSS to remove the blood cells and intestinal content until the buffer remained clear. Thereafter, the material was digested with 0.05 mg/mL collagenaseI (MP Biomedicals, United States) at 37°C under steady agitation for 20 min. The material was filtered and larger pieces were discarded, and then centrifuged at 800 rpm for 10 min. After discarding the supernatant, cell pellets were washed twice with HBSS at 800 rpm for 10 min, and resuspended in DMEM-F12 (GIBCO, United States) supplemented with 10% FBS (Crystalgen, United States), 1 × 10^7^ cells/well were seeded in 6 well plates and incubated at 37°C and 5% CO_2_. Only wells with over 80% cell confluency after 2 days of culturing were used for trials. The primary IECs were proved to have the same function with that *in vivo* (Supplementary Figure 1).

The cultured IECs were rinsed with HBSS for three times, starved in non-serum DMEM-F12 medium with 20 mM HEPES (Solarbio, China) for 2 h. For mRNA and protein expression analysis: IECs were starved for 2 h, treated with or without 3 mM acetate, 1 mM propionate and 1 mM butyrate for 24 h, respectively. In view of the limited IECs from one chicken embryo, the stimulating effect of acetate, propionate, and butyrate on GLP-1 secretion was evaluated individually. Then the cells were rinsed with HBSS for three times, and harvested for subsequent analysis. For GLP-1 secretion detection: IECs were treated for 2 h with SCFAs, with or without the MAPK inhibitors: the ERK-specific inhibitor (UO126, 10 μM), JNK inhibitor (SP600125, 20 μM), and p38 inhibitor (SB203580, 10 μM). Subsequently, the cell supernatants were collected and centrifuged for 10 min at 3,000 rpm. Total GLP-1 levels in cell supernatants were measured using High Sensitivity GLP-1 Active ELISA-Chemiluminescent (Merck Millipore, Germany) according to the reports from Nishimura et al. (2017).

### Primary Culture of Chicken Hepatocytes

Hepatocytes were prepared from freshly dissected liver tissues of 17-day-old SPF chick embryos. Livers were collected and washed with HBSS for three times. During this process, gallbladder, sarcolemma and connective tissue were carefully removed. Thereafter, livers were spliced into small pieces (about 1 mm^3^), digested with 0.01 mg/mL collagenase IV (Sigma, United States) for 5 min at 37°C, and gently blown with the disposable pipette for 5 min to disperse the cells. After filtration and centrifugation for 5 min at 1,000 rpm, the cells were collected and washed with HBSS for three times. Density gradient centrifugation was used to separate the hepatocytes from other cells, which was conducted in a layer with 60% Percoll (Sigma, United States). The cell suspension was loaded on the Percoll layer and centrifuged for 15 min at 3,000 rpm. The cells were collected and washed three times with HBSS. The separated cells were counted and seeded at a density of 1 × 10^6^ cells/mL, cultured in William’s E Medium (GIBCO, United States) supplemented with 10% FBS (Crystalgen, United States). Cells were incubated in a humidified incubator (Thermo Incubator, United States) at 37°C with 5% CO_2_ for 72 h. The medium was changed at 2 days intervals. Cells used in the present study have the same function with that *in vivo* (Supplementary Figure 2).

Chicken GLP-1 (sequence: HAEGTYTSDITSYLEGQA AKEFIAWLVNGRG, Honda, 2016), synthesized by Mimotopes (Hangzhou, China). The heptocytes were treated with 100 nM chicken GLP-1 for 24 h in the presence of 200 μM palmitic acid in the medium. Since GLP-1 is easily degraded, the heptocytes were also treated with 100 nM liraglutide (Selleck, United States) in the absence or presence of 100 nM exendin (9–39) (Aladdin, China), the inhibitor of GLP-1R. The cells were harvested for further analysis.

### Cell Viability

The cell viability under different treatments was determined by CCK-8 kit (Trans, China) at the wavelength of 450 nm.

### Oil Red O Staining

Cells were washed with cold phosphate buffered saline (PBS) and fixed in 10% paraformaldehyde for 30 min. Then the cells were stained for 30 min in a freshly diluted Oil Red O solution (Solarbio, China). After rinsed in distilled water, the cells were counter-stained for 2 min in Mayer’s Hematoxylin (Sigma-Aldrich, United States). The image of each group was photographed. Subsequently, the stained lipid droplets were extracted with isopropanol for quantification by measuring its absorbance at 490 nm.

### RNA Isolation and Quantitative Real-Time PCR Analysis

Total RNA from cultured cells, intestinal tract, liver and abdominal adipose tissues were prepared by the acid phenol method using Trizol reagent (Invitrogen, United States) according to the manufacturer’s instructions. 1.0 μg total RNA was reverse-transcribed into cDNA using the transcriptor first-strand cDNA synthesis kit (Roche, China). qPCR was conducted using FastStart Universal SYBR Green Master (Rox) reagents (Roche) to evaluate the relative mRNA expression. All the primers were designed by Primer 5, and standard curves and melting curves were performed to ensure the specificity and PCR efficiency. Each sample was amplified in duplicate, and GADPH was used as an internal control. Primers used for qRT-PCR were listed in Table 2.

**TABLE 2:** qRT-PCR primers.

**Gene**	**Sequence(5′–3′)**
FFAR2	Forward: AACGCCAACCTCAACAAGTC
FFAR2	Reverse: TGGGAGAAGTCATCGTAGCA
FFAR3	Forward: GAAGGTGGTTTGGGAGTGAA
FFAR3	Reverse: CAGAGGATTTGAGGCTGGAG
GADPH	Forward: CTACACACGGACACTTCAAG
GADPH	Reverse: ACAAACATGGGGGCATCAG
GLP-1R	Forward: GCTGAGAATGGCTGAGGAAC
GLP-1R	Reverse: CTTTGACTTGCTGTGCTCCA
ACC	Forward: AATGGCAGCTTTGGAGGTGT
ACC	Reverse: TCTGTTTGGGTGGGAGGTG
FAS	Forward: TCCTTGGTGTTCGTGACG
FAS	Reverse: CGCAGTTTGTTGATGGTGAG
SREBP-1c	Forward: GCCCTCTGTGCCTTTGTCTTC
SREBP-1c	Reverse: ACTCAGCCATGATGCTTCTTCC
LPL	Forward: CAGTGCAACTTCAACCATACCA
LPL	Reverse: AACCAGCCAGTCCACAACAA
PPARG	Forward: TCCTTCCCTCTGACCAAA
PPARG	Reverse: AATCTCCTGCACTGCCTC
Adipo	Forward: TCACCTACGACCAGTTCCA
Adipo	Reverse: CCCGTTGTTGTTGCCCTC
Fabp4	Forward: TGAAGCAGGTGCAGAAGT
Fabp4	Reverse: CAGTCCCACATGAAGACG

### Western Blot Analysis

Total protein extracts from cultured cell lysates or tissue samples were prepared by homogenization in RIPA buffer (1% Nonidet P-40, 0.5% sodium deoxycholate, and 0.1% sodium dodecyl sulfate in PBS) supplemented with protease inhibitor cocktail (Sigma-Aldrich, Canada) and phosphatase inhibitor cocktail (Fdbio, China). Cell and tissue homogenates were centrifuged at 12,000 × *g* and 4°C for 10 min. The protein content of the supernatants was determined using the BCA protein assaykit (Beyotime, China). Total protein (30 μg) was separated by SDS-PAGE and transferred to PVDF membranes (Millipore, Germany) using a transfer apparatus (Bio-Rad, United States). The membranes were blocked with blocking buffer (Beyotime, China) at room temperature for 1 h, then incubated with anti-phospho-P38 [#4511T, anti-rabbit, Cell Signaling Technology (CST), United States], anti-P38 (#9212S, anti-rabbit, CST), anti-phospho-JNK (#4668S, anti-rabbit, CST), anti-JNK (#928, anti-rabbit, CST), anti-phospho-ERK (#9101S, anti-rabbit, CST), and anti-ERK (#9102S, anti-rabbit, CST), anti-phospho-ACC (#3661, anti-rabbit, CST), anti-ACC (#3662, anti-rabbit, CST), anti-phospho-AMPK (anti-rabbit, CST) or anti-AMPK (anti-rabbit, CST) primary antibodies overnight at 4°C. Thereafter, the membrane was incubated with the corresponding horseradish peroxidase-conjugated secondary antibody (Beyotime, China) at 4°C for 4 h. Tubulin was used as an internal control for MAPK pathway assay, and β-actin was used as an internal control for other protein expression assays. The protein–antibody complexes were detected with the ECL Plus A and B (Beyotime, China), and the results were quantified using the Fusion FX software (Vilber, France).

### Statistical Analysis

The data were expressed as mean ± SE and analyzed by one-way ANOVA with SAS software. Differences between means were evaluated using Duncan’s significant difference tests. *p* < 0.05 was considered as statistically significant.

## Results

### PB and GG Treatments Suppressed Lipid Synthesis and Accumulation in the Liver and Abdominal Fat Tissues

**FIGURE 1:**
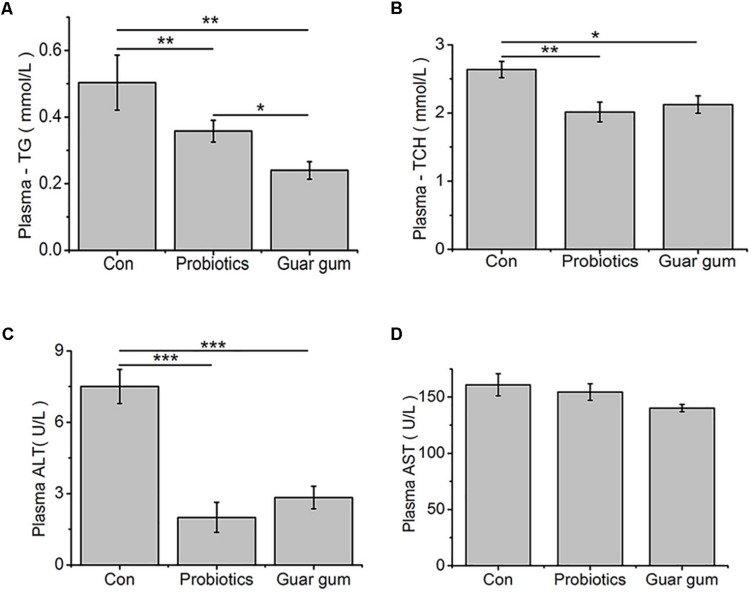
Effect of probiotics and Guar gum supplementations on plasma parameters of 21-day-old broilers. **(A)** TG; **(B)** TCH; **(C)** ALT activity; **(D)** AST activity. Data were presented as Mean ± SE (*n* = 8). ^∗^*p* < 0.05, ^∗∗^*p* < 0.01, and ^∗∗∗^*p* < 0.001.

The GG-treated chickens had the lowest plasma TG concentration while the control birds had the highest one (*p* < 0.05, Figure 1A). Compared to the control, the plasma TCH content was decreased by both PB (*p* < 0.01) and GG (*p* < 0.05) treatments (Figure 1B). Plasma activity of ALT was decreased in both PB- and GG-treated chickens comparing with control (*p* < 0.001), while AST was not changed (*p* > 0.05, Figures 1C,D).

**FIGURE 2:**
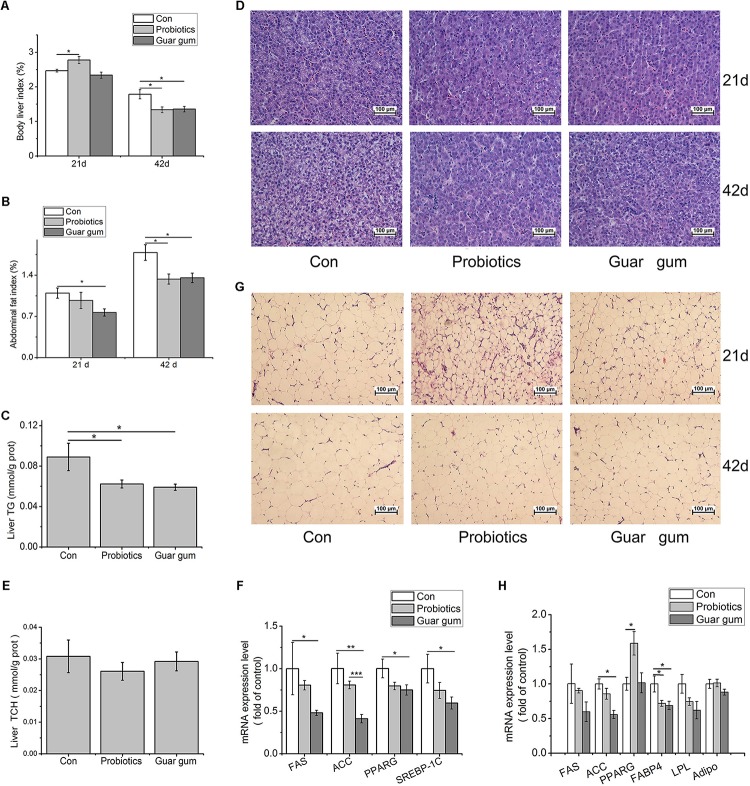
Effects of probiotics and Guar gum supplementation on fat deposition in broilers. **(A)** Liver index at day 21 and days 42 (*n* = 10); **(B)** Abdominal fat index at day 21 and day 42 (*n* = 10); **(C)** TG contents in the liver (*n* = 10); **(D)** H&E staining of the liver slides at day 21 and day 42 (original magnification: ×200, *n* = 6); **(E)** TCH contents in the liver (*n* = 10); **(F)** the mRNA expression levels of FAS, ACC, PPARG, and SREBP-1c in the liver at day 21 (*n* = 8); **(G)** H&E staining of the abdominal fat slides at day 21 and day 42 (original magnification: ×200, *n* = 6); (H) the mRNA expression levels of FAS, ACC, PPARG, FABP4, LPL, Adipo (adiponection) in abdominal adipose tissue at day 21 (*n* = 8). SAS analysis followed by *t*- test. Data were presented as Mean ± SE. ^∗^*p* < 0.05, ^∗∗^*p* < 0.01, and ^∗∗∗^*p* < 0.001.

At day 21, the liver index was significant higher in the PB group compared with control group (*p* < 0.05, Figure 2A). The abdominal fat index was markedly lower in GG-treated chickens than that in the control (*p* < 0.05, Figure 2B). At day 42, in contrast to the control, both liver and abdominal fat indexes were decreased by PB and GG treatments (*p* < 0.05) (Figures 2A,B). Compared to control, chickens in PB and GG groups showed decreased TG content (*p* < 0.05, Figure 2C) and alleviated fatty infiltration in liver at both 21 and 42 days of age (Figure 2D). The hepatic TCH contents, however, was not influenced by either dietary treatment (*p* > 0.05, Figure 2E).

To further investigate the molecular changes under the decreased lipid contents in PB- and GG-treated chickens, key mediators involved in the *de novo* lipogenesis in liver were detected. Compared to control, the mRNA expression levels of fatty acid synthase (FAS), peroxisome proliferator-activated receptor-γ (PPARG), and sterol regulatory element-binding protein (SREBP)-1c were all down-regulated by GG treatment (*p* < 0.05, Figure 2F). ACC expression was highly suppressed in GG group compared with control (*p* < 0.001). In PB-chickens, the mRNA expression levels of FAS, PPARG and SREBP-1 showed the same trends with that in GG-chickens, however, only the ACC expression was highly suppressed compared with control (*p* < 0.01).

In abdominal fat tissues, the average adipocyte size was reduced in PB- and GG-chickens versus control (Figure 2G). The mRNA levels of ACC and fatty acid binding protein 4 (FABP4) in GG-treated chickens were statistically decreased compared to control (*p* < 0.05, Figure 2H). In contrast, PB treatment increased PPARG while decreased FABP4 transcription comparing with control (*p* < 0.05).

### PB and GG Treatments Changed the Cecal Microbiota Diversity

**FIGURE 3:**
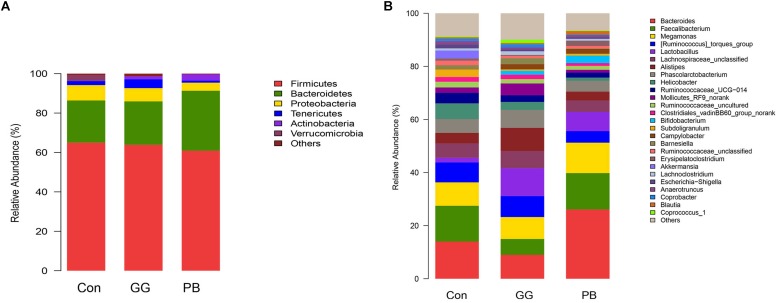
Probiotics and guar gum affect cecal bacteria structure. Relative abundance of bacteria population in cecal contents of Control, PB and GG groups at 21 days of age. Sequences were analyzed by using Illumina MiSeq System. **(A)** Phylum level; **(B)** Genus level (*n* = 8).

Compared to the control group, GG did not change the relative abundances of *Firmicutes* and *Bacteroidetes* at the phylum level, while PB decreased the relative abundance of *Firmicutes* and increased the relative abundance of *Bacteroidetes* (Figure 3). There was a decrease in the relative abundances of *Proteobacteria* in both PB and GG groups. The minor taxonomic groups such as *Actinobacteria* and *Verrucomicrobia* showed the same trends in PB and GG treatments. In line with that at the phylum level, minor taxonomic groups of cecal microbiota in PB and GG groups presented the same trends and showed significant differences with that in control group. Compared with the control, the *Escherichia–Shigella*, *Anaerotruncus*, *Akkermansia*, *Ruminococcaceae_uncultured*, *Subdoligranulum*, *Ruminococcaseae* and *Helicobacter* presented much lower abundance in both PB and GG groups, while *Bifidobacterium*, *Campylobacter* and *Lactobacillus* were significantly higher in PB and GG groups (Figure 3).

### PB and GG Treatments Are Associated With Altered Cecal SCFAs Concentrations

**FIGURE 4:**
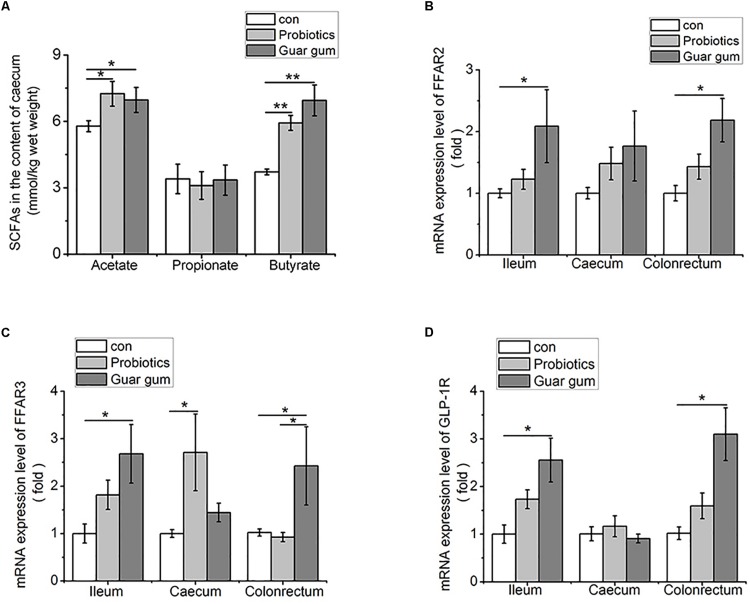
Higher contents of SCFAs increase FFARs and GLP-1R expression in the intestine of both PB and GG groups at 21 days of age. **(A)** SCFAs in the cecum contents (mmol/kg wet weight) of chickens in control, PB and GG groups at 21 days of age (*n* = 10 in control and PB groups; *n* = 8 in GG group); **(B–D)** effect of probiotics and guar gum on mRNA expression levels of FFAR2 **(B)**, FFAR3 **(C)**, and GLP-1R **(D)** in the intestine of chickens at 21 days of age (*n* = 8). Data were presented as Mean ± SE, ^∗^*p* < 0.05 and ^∗∗^*p* < 0.01.

Given the cecal microbiota altered in a certain degree of similarity under the PB and GG treatments, we further investigated the concentrations and relative distributions of the major SCFAs (acetic, propionic and butyric acids) in cecum. Compared to the control, cecal acetate concentrations were increased in both PB and GG groups (*p* < 0.05, Figure 4A), as well as butyrate levels in PB and GG groups were greatly increased (*p* < 0.01). However, the propionate content was not influenced by PB or GG treatment (*p* > 0.05).

### PB and GG Treatments Upregulated the Expression of FFARs and GLP-1R

To evaluate the responses to increased SCFAs, FFAR2/3 and GLP-1R mRNA expression levels in the ileum, cecum and colonrectum were analyzed. Comparing with control, the mRNA levels of FFAR2, FFAR3 and GLP-1R were upregulated by GG treatment in ileum, ceacum and colonrectum (*p* < 0.05), but not in cecum (*p* > 0.05, Figures 4B–D). Except for FFAR3 mRNA level was increased by PB treatment in cecum (*p* < 0.05) (Figure 4C), no significant influence was observed for FFAR2, FFAR3, and GLP-1R mRNA levels in other tissues by PB treatment.

### SCFAs Induced GLP-1 Secretion via Activating MAPK Pathways in IECs

**FIGURE 5:**
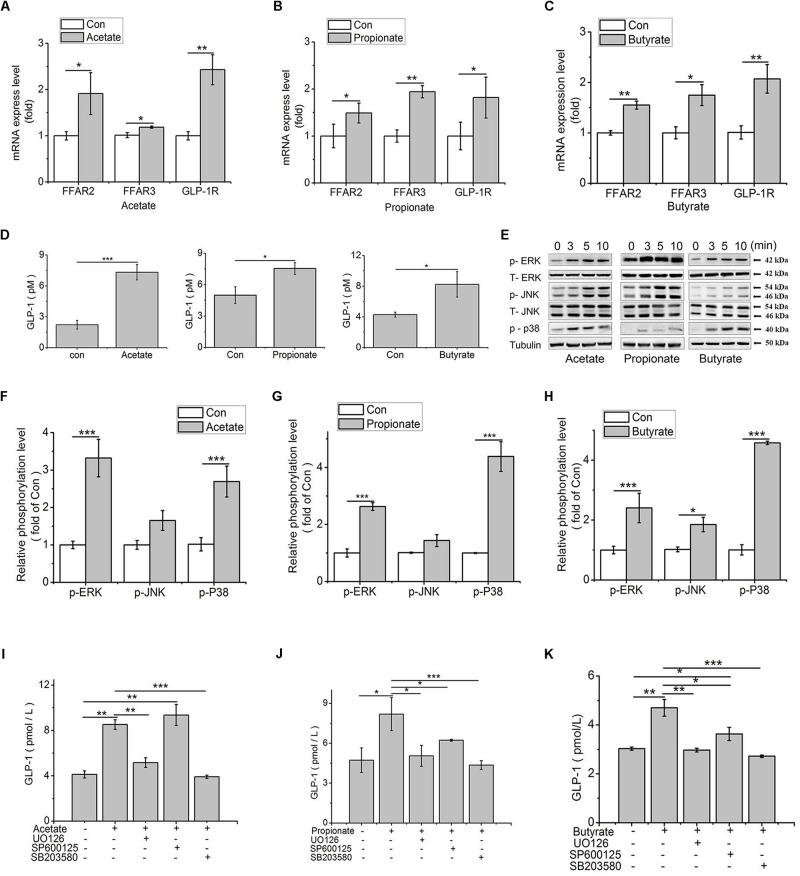
SCFAs contribute to GLP-1 secretion via MAPK pathways in IECs. **(A–C)** Transcription levels of FFAR2 **(A)**, FFAR3 **(B)**, and GLP-1R **(C)** were assessed by qPCR in primary IECs treated with 3 mM acetate, 1 mM propionate, and 1 mM butyrate for 24 h (*n* = 8); **(D)** GLP-1 concentrations in the IECs culture medium after treatments of 3 mM acetate, 1 mM propionate, and 1 mM butyrate for 2 h, respectively (*n* = 8); **(E)** SCFAs-mediated activation of ERK, JNK and p38MAPK in IECs treated with 3 mM acetate, 1 mM propionate, and 1 mM butyrate. Intracellular levels of p-ERK, p-JNK, and p-p38 were analyzed by western blotting. Relative phosphorylation levels were calculated by p-p38, and p-ERK/ERK and pJNK/JNK, and which were normalized to control (*n* = 4); **(F–H)** Quantity of p-ERK, p-JNK, and p38MAPK at 5 min after treatment of SCFAs: **(F)** Acetate, **(G)** Propionate, and **(H)** Butyrate; **(I–K)** MAPK inhibitors block SCFA-induced increase of GLP-1 secretion. IECs were treated with 3 mM acetate, 1 mM propionate, and 1 mM butyrate, with or without the ERK-specific inhibitor (UO126, 10 μM), JNK inhibitor (SP600125, 20 μM), and p38 inhibitor (SB203580, 10 μM), respectively. GLP-1 concentration was detected by high sensitivity GLP-1 Active ELISA-Chemiluminescent kit (*n* = 6): **(I)** Acetate, **(G)** Propionate, **(H)** Butyrate. Data were presented as mean ± SE. ^∗^*p* < 0.05, ^∗∗^*p* < 0.01, and ^∗∗∗^*p* < 0.001.

To verify whether and how SCFAs affected the secretion of GLP-1, the mRNA expression of FFAR2/3 and GLP-1R were analyzed in SCFAs-treated IECs. Compared to the control, the expression levels of FFAR2, FFAR3, and GLP-1R were significantly up-regulated by acetate (*p* < 0.05, *p* < 0.05, *p* < 0.01), propionate (*p* < 0.05, *p* < 0.01, *p* < 0.01), and butyrate (*p* < 0.01, *p* < 0.05, *p* < 0.01; Figures 5A–C) in IECs. Additionally, the GLP-1 content in culture medium was detected. Results showed that the GLP-1 content was increased by acetate (*p* < 0.001), propionate (*p* < 0.05), and butyrate (*p* < 0.05) (Figure 5D).

According to the published literatures, FFARs can activate ERK signal pathway. To elucidate how the signal is transduced from FFARs to GLP-1, the influence of SCFAs on MAPK signal pathways were detected. Results showed that the phosphorylation levels of ERK and p38 were all significantly increased (*p* < 0.001) by acetate, propionate, and butyrate treatment (*p* < 0.001). The phosphorylation of JNK was only significantly upregulated by butyrate (*p* < 0.05) (Figures 5E–H).

To confirm the signal transduction pathways, we used pharmacological inhibitors of MEK1/2, p38 MAPK and JNK to evaluate the role of MAPKs in the release of GLP-1 in IECs. Compared to control, acetate treatment increased GLP-1 concentration (*p* < 0.01), which was abolished in the presence of UO126 (*p* < 0.01) and SB203580 (*p* < 0.001), the inhibitors of ERK and p38, but not influenced by SP600125, the inhibitor of JNK (Figure 5I). In contrast, the stimulating effect of propionate on GLP-1 secretion (*p* < 0.05) was inhibited by UO126 (*p* < 0.05), SB203580 (*p* < 0.05), and SP600125 (*p* < 0.001, Figure 5J). Similarly, UO126 (*p* < 0.01) and SB203580 (*p* < 0.001) arrested the stimulating effect of butyrate on GLP-1 secretion (*p* < 0.01). Compared to control and butyrate treatment, however, SP600125 treatment increased and decreased, respectively, the GLP-1 secretion (*p* < 0.05, Figure 5K).

### GLP-1 and Its Analog Liraglutide Suppressed Lipid Accumulation Through AMPK/ACC Phosphorylation in Primary Hepatocytes

**FIGURE 6:**
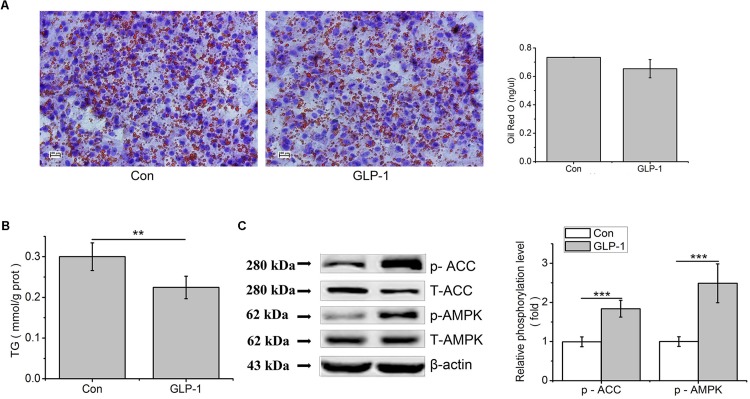
GLP-1 reduces lipid accumulation in the primary hepatocytes. Hepatocytes were stimulated with 100 nM GLP-1 for 24 h. **(A)** Oil red O staining (original magnification: ×200, *n* = 6). **(B)** TG content in hepatocytes was measured and normalized to the total protein. **(C)** GLP-1 increased the phosphorylation of AMPK and ACC. Intracellular levels of p-AMPK and p-ACC were analyzed by western blotting. Relative phosphorylation levels were calculated by pAMPK/AMPK and pACC/ACC, which were normalized to control. Significant comparisons were calculated by SAS with a post *t*-test. Data were presented as mean ± SE (*n* = 6). ^∗∗^*p* < 0.01 and ^∗∗∗^*p* < 0.001.

In order to study the effect of GLP-1 on lipid synthesis, we examined the consequences of GLP-1 and its analog on hepatic lipid accumulation. Results showed that, in primary hepatocytes, the lipid accumulation that stained with Oil Red O and TG contents were decreased by GLP-1 treatment, compared to the control (*p* < 0.01, Figures 6A,B). Compared to the control, phosphorylation levels of AMPK and ACC were strongly increased by GLP-1 (*p* < 0.001, Figure 6C).

**FIGURE 7:**
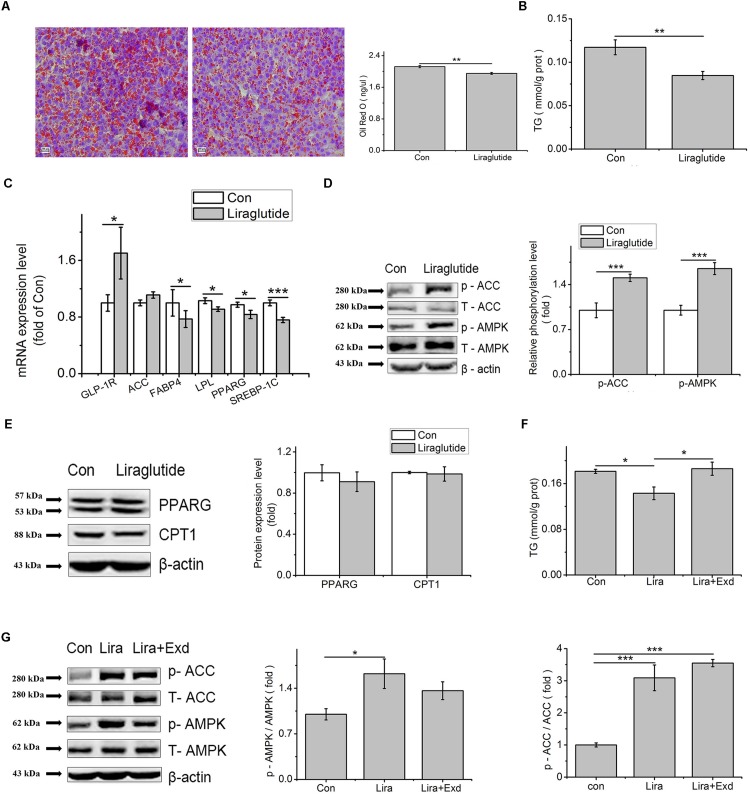
Liraglutide can mimic the effect of GLP-1 on lipid accumulation in the primary hepatocytes. Hepatocytes were treated with 100 nM liraglutide for 24 h. **(A)** Oil red O staining (original magnification: ×200, *n* = 6); **(B)** TG content in hepatocytes, normalized to the total protein (*n* = 6); **(C)** mRNA expression levels of GLP-1R, ACC, FABP4, LPL, PPARG, and SREBP-1c after 24 h treatment of liraglutide (*n* = 6); **(D)** The phosphorylation levels of pAMPK and pACC, normalized to control (*n* = 4); **(E)** The expression of PPARG and CPT1 (*n* = 4); **(F)** Effect of exendin on liraglutide-reduced TG content; **(G)** Exendin inhibited the phosphorylation of AMPK and ACC, normalized to control. Data were presented as mean ± SE. ^∗^*p* < 0.05, ^∗∗^*p* < 0.01, ^∗∗∗^*p* < 0.001.

Liraglutide treatment decreased the Oil Red O and TG content in the primary hepatocytes, compared to control (*p* < 0.01, Figures 7A,B). Liraglutide upregulated the transcription level of GLP-1R (*p* < 0.01), while down regulated the mRNA levels of PPARG (*p* < 0.05), FABP4 (*p* < 0.05), LPL (*p* < 0.05), and SREBP-1C (*p* < 0.001) (Figure 7C).

Compared to control, liraglutide increased the phosphorylated AMPK (*p* < 0.001) and ACC (*p* < 0.001, Figure 7D), but had no influence on PPARG and carnitine palmityl transferase I (CPT1) protein expression (*p* > 0.05, Figure 7E). In comparation with control, the decreased TG content caused by liraglutide was restored by exendin (9-39) (Figure 7F). Although there was no significant difference, the phosphorylation level of AMPK in exendin (9-39) + liraglutide treatment was deceased comparing with liraglutide treatment (*p* > 0.05), and tended to the level of the control group (*p* > 0.05). Compared with liraglutide treatment, the phosphorylation level of ACC, however, was not changed by exendin (9–39) (*p* > 0.05) (Figure 7G).

## Discussion

### Altered Gut Microbiota Contributes to the Increased SCFAs and Decreased Lipid Accumulation

Gut microbes have been shown to regulate host physiology, metabolism, and energy storage (Everard and Cani, 2013; Villanueva-Millán et al., 2015). Altered gut microbial composition have been observed in animals and humans with metabolic syndrome (Geurts et al., 2011; Velasquez, 2018). John and Mullin (2016) indicated that obesity accompanies with the increase of *Firmicutes* and decrease of *Bacteroidetes*. Though large amounts of correlation research have been reported, the linkage of gut microbiome with metabolism still remains largely to be elucidated.

This study indicated that both PB and GG could change the cecal microbiota structure and diversity. In line with previous studies in human and rodents (Ley et al., 2005; Turnbaugh et al., 2006), the decreased proportion of phylum *Firmicutes* and increased proportion of phylum *Bacteroidetes* were observed in PB group. At the genus level, the relative abundance of *Lactobacillus*, *Bifidobacteria*, and *Campylobacter* were increased in both PB and GG groups. In broilers, studies have shown that probiotics and prebiotics could decrease the pH and increase the numbers of *Lactobacillus* and *Bifidobacteria* in caecum (Al-Khalaifa et al., 2019). Guar gum has also been proved to stimulate *Bifidobacterium* and butyrate-producing bacteria in large intestine (Ohashi et al., 2015). *Campylobacter* is ubiquitous in nature and forms a part of the natural intestinal microbiota of poultry, while *Campylobacter jejuni* is one of the most common bacterial causes of gastroenteritis in human. The field study indicates that dietary probiotic supplementation for broiler chickens is capable to reduce the extent of *Campylobacter* spp. invasion in the gastrointestinal tract of birds (Smialek et al., 2018). The probiotics outcompete *Campylobacter jejuni* through different mechanisms of actions, particularly through increased velocity to reach the predilection sites, competitive exclusion and occupation of adhesions sites on the IECs, alteration of luminal pH, production of bacteriocins, strengthening of tight junction proteins and modulation of immune system, quorum sensing, and enhanced bacterial cross-talk (Mohan, 2015). In the present study, we observed the proportion of *Campylobacter* was increased in PB and GG groups. However, this result should be interpreted with caution as the absolute amount of *Campylobacter* was not measured. The dietary supplementation of the PB and GG on dimination of *Campylobacter* needs to be further investigated. *Lactobacilli* and *Bifidobacteria* were indicated to produce SCFAs during the fermentation of carbohydrates (Macfarlane and Macfarlane, 2003). Studies have shown that *Lactobacillus salivarius* and *L. agilis* increased propionate and butyrate contents in caecum of chickens (Meimandipour et al., 2010), *Lactobacillus* and *Bifidobacterium* increased the commensal metabolite butyrate (Liang et al., 2018). Consistently, supplementation with PB and GG leads to increased cecal contents of acetate and butyrate in this study. However, propionate in cecum in both PB and GG groups had no differences with that in control group. This may be due to the different experimental periods or the different feeds fed to chickens.

Associating with higher cecal acetate and butyrate contents were detected in PB- and GG- treated chickens, lower hepatic and plasma TG/TCH concentrations, and reduced abdominal fat ratio were observed in the two groups. Similar effects associated with probiotics and guar gum were observed in mammals. Wang et al. (2017) demonstrated that probiotic *Lactobacillus johnsonii* decreased fat deposition by improving lipid metabolism in broilers (Wang et al., 2017). Guar gum can be digested and produces SCFAs in the hindgut, and prevent or reverse body weight gain in rodents and humans (Krotkiewski, 1984; Butt et al., 2007; den Besten et al., 2015). Other works demonstrated that dietary supplementation of acetate, propionate, and butyrate inhibits lipolysis and *de novo* lipogenesis and protects against HFD-induced obesity (Ge et al., 2008; Lin et al., 2012; Heimann et al., 2014; Chambers et al., 2015). Collectively, these finding gave evidence that SCFAs may be a main mediator for the lipid regulation of probiotics or guar gum, and liver is a vital target of gut microbial regulation on fat metabolism in chickens.

### SCFAs Induce the Secretion of GLP-1 via MAPK Pathways

Although the intracellular mechanism is not fully understood, luminal SCFAs are expected to stimulate FFAR2 and/or FFAR3 located on the colonic L cells and induce GLP-1 release in mammals (Kaji et al., 2014). In the present study, the increased contents of SCFAs were accompanied with higher expression levels of FFARs and GLP-1R in chicken intestines. Hudson et al. (2012) showed that FFAR2 and FFAR3 respond to acetate and butyrate at the same level, while FFAR3 is more sensitive to propionate than FFAR2 in mice (Brown et al., 2003). A slightly different nature from that in mice, our results indicated that both chicken FFAR2 and FFAR3 responded to propionate and butyrate at the same level, and FFAR3 was a little more sensitive than FFAR2 *in vitro*. Meanwhile, FFAR2 was much more sensitive to acetate than FFAR3 in primary IECs.

In primary cultured IECs, all three SCFAs increased the release of GLP-1. It has been demonstrated that luminal and especially vascular infusion of acetate and butyrate significantly increase colonic GLP-1 secretion, while propionate has no influence on GLP-1 secretion either administered luminally or vascularly in rat (Christiansen et al., 2018). In contrast, although the effect of propionate on GLP-1 secretion is the weakest among the three SCFAs, propionate also could significantly promote the secretion of GLP-1 in chicken IECs. Meanwhile, GLP-1 secretion showed a threefold increase by acetate and a twofold increase by butyrate respectively. FFAR2 activation is suggested to predominate over FFAR3 signaling induced by SCFAs with regards to increased gut hormone release (Brown et al., 2003; Psichas et al., 2015). Tolhurst et al. (2012) reported that SCFAs stimulate GLP-1 secretion via FFAR2 in mixed colonic cultures in mice (Psichas et al., 2015), which suggested that the different GLP-1 secretion may be related to the varied activation of FFAR2 under stimulation of acetate, propionate and butyrate. In this study, we found that acetate had the strongest stimulating effect on FFAR2, followed by butyrate and propionate in IECs. In the present study, the effect of acetate, propionate, and butyrate on GLP-1 secretion was respectively determined with different batches of IECs separated from chicken embryos, which should be responsible for the varied GLP-1 basal value in control treatment.

FFAR2 and FFAR3 are G protein coupled receptors (GPCRs). In human and rodents, the coupling of FFAR2 and FFAR3 to ERK1/2 was confirmed under the stimulation of SCFAs (Le Poul et al., 2003; Seljeset and Siehler, 2012). Yonezawa et al. (2007) showed that all three SCFAs rapidly and selectively activated p38 MAPK in MCF-7 cells. JNK can also be activated by acetate, propionate or butyrate in HEK293 cells, but has rarely been studied due to its lower activation level compared to ERK (Seljeset and Siehler, 2012). It is well known that ERK is a major regulator of cell proliferation, whereas JNK and p38MAPK are involved in stress signaling and many inflammatory processes (Huang et al., 2009). Does MAPK pathway participate in the SCFAs mediated GLP-1 secretion and involve in lipid metabolism? In the animal model of this study, the chickens were fed with a diet supplemented with PB or GG and the change in MAPK pathway cannot be ascribed to the effect of SCFAs. Hence, we tested the hypothesis in IECs from chicken embryos, which has been proved to have the same characteristics as that in the gut tissues from chickens after hatching and have been extensively used in previous studies (Yuan et al., 2015; Lin et al., 2016). Our present study showed that ERK was stimulated by acetate for 3.5 folds, by propionate for 3 folds, and by butyrate for 2.5 folds, compared to the control. In contrast, significant JNK activation was only detected under butyrate treatment. This result was in agreement with the study that ERK1/2 phosphorylation level mediated by FFAR2 and FFAR3 were extremely higher with over 3–4 fold to the control, while activation of JNK1/3 was weak (Seljeset and Siehler, 2012). The activated p38 MAPK was observed in all three SCFAs treatments, which was disagree with the work in parental HEK293 cells, where the activation of p38 MAPK was weak under both FFAR2 and FFAR3 stimulators (Seljeset and Siehler, 2012). Additionally, in accordance with highly activated ERK and p38 MAPK under the stimulation of all three SCFAs, the GLP-1 secretion was reduced to the basal level by inhibitors of ERK1/2 and p38MAPK. In contrast, the acetate mediated GLP-1 secretion was not sensitive under the inhibitor of JNK. These findings suggest that ERK and p38 MAPK pathways are mainly involved in SCFAs-induced GLP-1 secretion in chickens, which are different from that in mammals. This conclusion, however, needs to be further consolidated by *in vivo* study.

### GLP-1 and Its Analog Liraglutide Decrease Hepatic Fat Synthesis Through Phosphorylation of AMPK and ACC

Short chain fatty acids have been shown to stimulate the gut hormone GLP-1 release (den Besten et al., 2015). Treatments with GLP-1 or GLP-1 agonists result in a reduced body weight gain, a decreased fat deposition (Blaut, 2015; Chambers et al., 2015; Petit and Vergès, 2017), and improved liver histology (Cuthbertson et al., 2012; Vilsbøll et al., 2012; Nishimura et al., 2017). It has been demonstrated that GLP-1 reduces body weight gain and fat deposition by suppressed food intake (Blaut, 2015). In this study, we found that both GLP-1 and liraglutide could decrease lipid accumulation in the primary cultured hepatocytes, indicating that liver was an important target organ for GLP-1, and GLP-1 could directly regulate lipid metabolism in broiler chickens. In *ob/ob* mice, 60 days of treatment with GLP-1R agonist significantly reduced weight gain and hepatic lipid content, suggesting that GLP-1 has a direct effect on the fat metabolism in liver and the GLP-1-treated hepatocytes showed elevated cAMP production as well as reduced mRNA expression of genes associated with fatty acid synthesis (Ding et al., 2006). Our study showed that GLP-1 and its analog liraglutide significantly increased the phosphorylation of AMPK and ACC, whereas had no effect on PPARG and CPT1 at the protein level in chicken primary hepatocytes. AMPK plays a key role in regulating energy metabolism. Activated AMPK can phosphorylate and inactivate ACC which leads to a decrease in fatty acid synthesis (Hardie and Pan, 2002; Ben-Shlomo et al., 2011), as well as down-regulates transcription factors and enzymes associated with lipid metabolism, such as SREBP-1c and FAS (Viollet et al., 2006; Postic and Girard, 2008; Viollet et al., 2009). Additionally, our results showed that liraglutide reduced the key regulators of *de novo* lipogenesis, such as PPARG, SREBP-1C and LPL at the transcription level. This indicates that the decreased lipid accumulation is at least partially due to the inactivation of ACC which phosphorylated by p-AMPK and the decreased gene expression related to *de novo* lipogenesis. In the present study, the effect of GLP-1 was further validated by the use of exendin (9–39), a competitive GLP-1 receptor antagonist. The reduced TG accumulation in hepatocytes by liraglutide was reversed in the presence of exendin (9–39), indicating that the blockage of GLP-1R partially suppresses the stimulating effect of liraglutide. This result was in line with the observation that the augmented phosphorylation of AMPK by liraglutide was partially reduced by exendin (9–39). Hence, the present result implies that exendin (9–39) can partially suppress the stimulating effect of liraglutide in the present condition.

## Conclusion

Our results hinted that the altered gut microbial structure leads to elevated production of SCFAs, which induce the enhanced secretion of GLP-1 in IECs via MAPK pathways. GLP-1 further reduces hepatic fat synthesis by activating AMPK/ACC pathway in chickens.

## Data Availability Statement

All datasets generated for this study are included in the manuscript/Supplementary Files.

## Ethics Statement

The animal study was reviewed and approved by the Institutional Animal Care and Use Committee at College of Animal Science of Shandong Agriculture University.

## Author Contributions

J-MZ and HL designed the research. J-MZ performed and analyzed all the experiments, and drafted, edited, and revised the manuscript. Y-SS, L-QZ, T-TC, and M-NF helped in performing and analyzing the animal experiments. H-CJ and J-PZ helped in designing the animal studies. X-JW developed the key methodologies for SCFAs analysis. F-CL helped in designing the cell experiments. H-FL and HL interpreted the results, and edited, revised, and approved the final version of the manuscript.

## Conflict of Interest

Y-SS, T-TC, and M-NF were employed by company Shandong Baolai-Leelai Bioengineering Co., Ltd. The remaining authors declare that the research was conducted in the absence of any commercial or financial relationships that could be construed as a potential conflict of interest.
