# Supplementary figures and images for: SCFAs-Induced GLP-1 Secretion Links the Regulation of Gut Microbiome on Hepatic Lipogenesis in Chickens
Source: Front Microbiol. 2019 Sep 26;10:2176. doi: 10.3389/fmicb.2019.02176 (PMC6775471; doi:10.3389/fmicb.2019.02176)

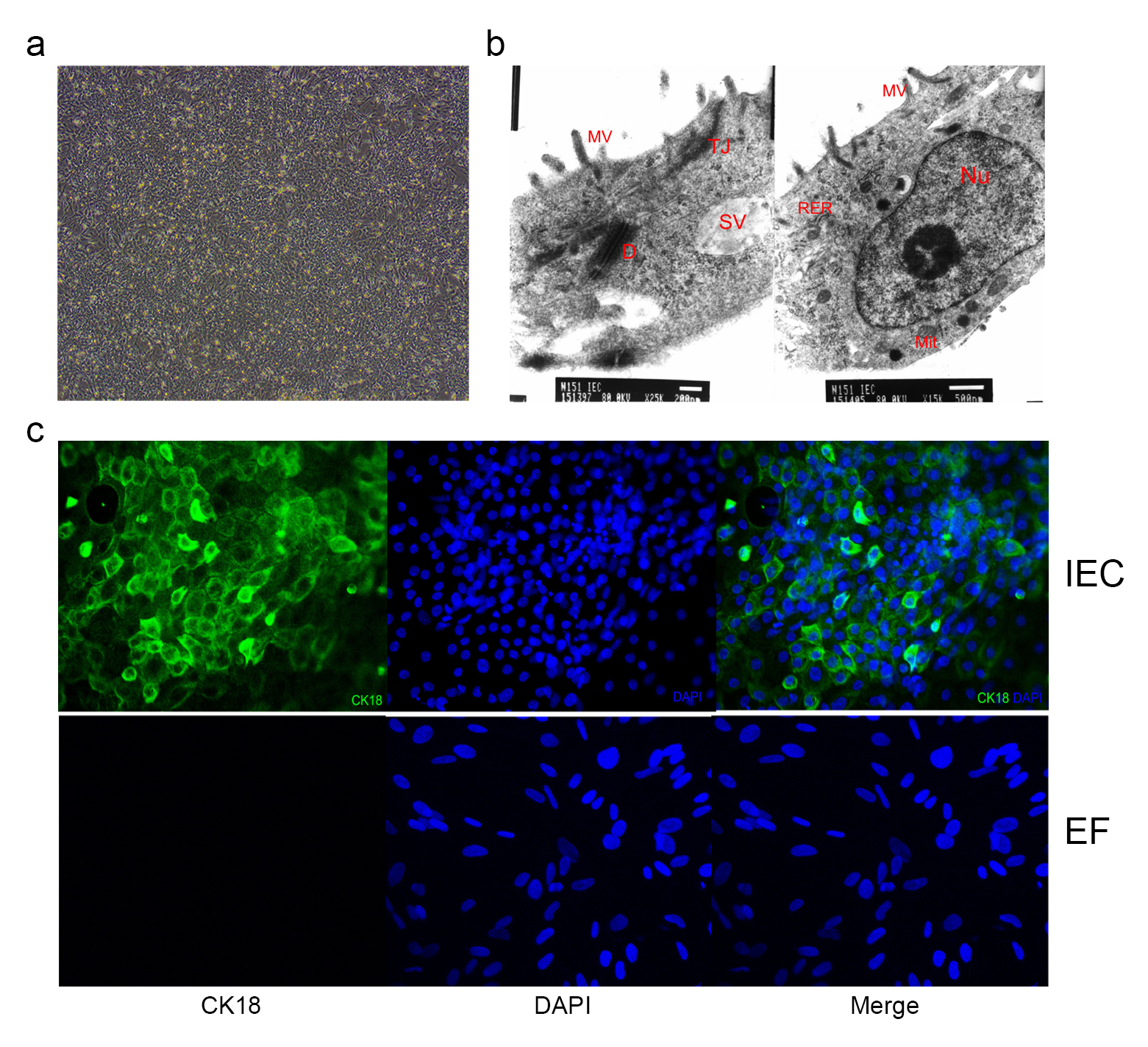

Supplement: Supplementary file 1 [file Image_1.JPEG]

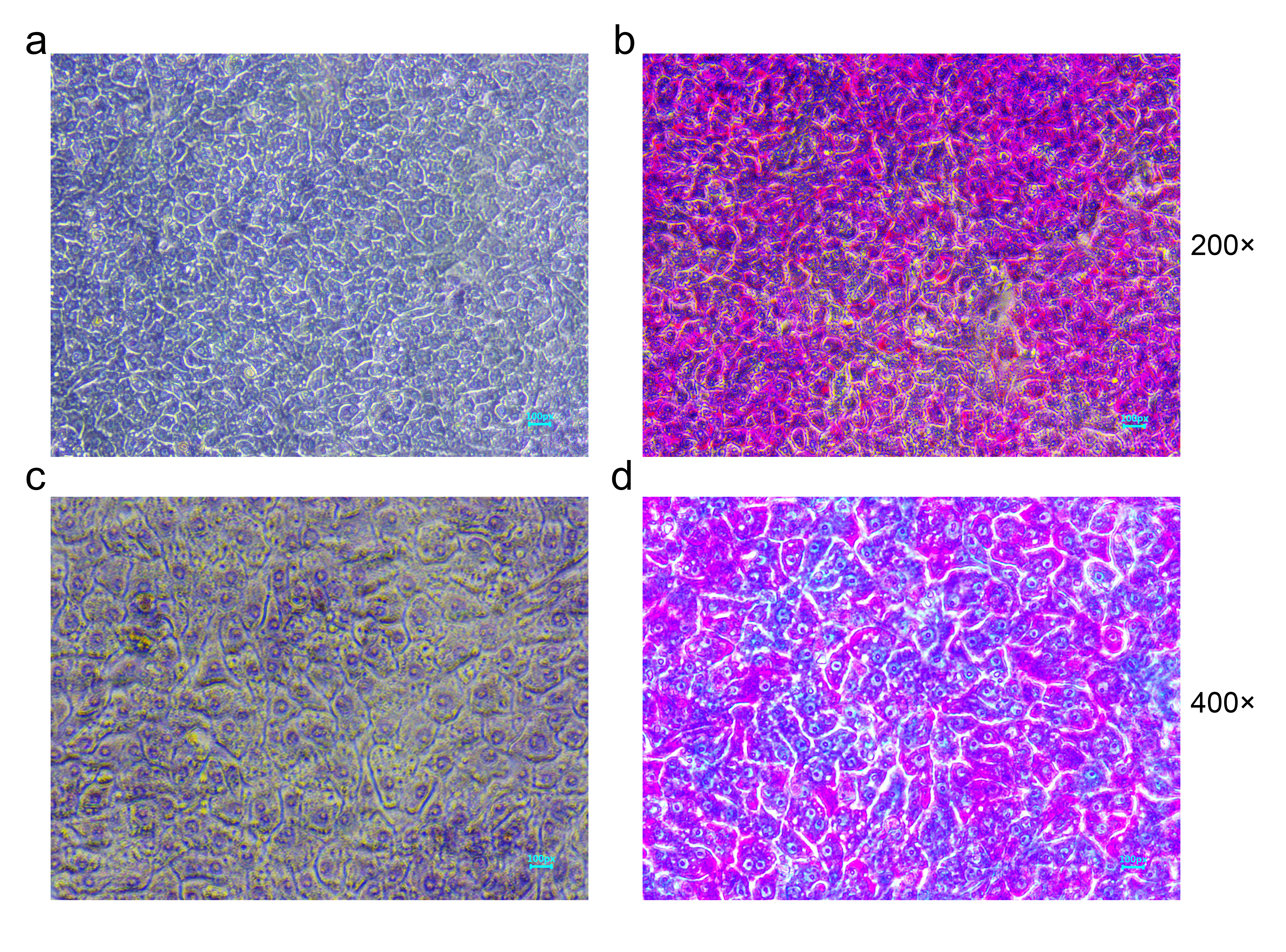

Supplement: Supplementary file 2 [file Image_2.JPEG]
