# Supplementary material for: SCFAs-Induced GLP-1 Secretion Links the Regulation of Gut Microbiome on Hepatic Lipogenesis in Chickens
Source: Front Microbiol. 2019 Sep 26;10:2176. doi: 10.3389/fmicb.2019.02176 (PMC6775471; doi:10.3389/fmicb.2019.02176)
Supplement: Supplementary file 3 [file Table_1.DOCX]

**The identification of the IECs and hepatocytes**

***The identification of the IECs***

As shown in supplementary Fig 1. a, after 48 hours of incubation, typical paving stone-like cell morphology can be observed in cultivated primary embryo intestinal epithelial cells (IECs) under a microscope. The unique microstructure of microvillus, desmosomes, and tight junctions in IECs could be observed by transmission electron microscope (Supplementary Fig 1. b). Furthermore, immunofluorescence assay was conducted to detect the expression of cytokeratin 18 (CK18) which is a specific marker protein of epithelial cells in IECs. The green fluorescence was detected in the cytoplasm of cultured IECs, while no green fluorescence was detected in esophageal fibroblasts the negative control (Supplementary Fig 1. c).

***The identification of the hepatocytes***

After 72h cultivation, the chicken primary embryo hepatocytes were verified by using periodic acid-Schiff (PAS) (Supplementary Fig 2). typical morphology of hepatocytes was observed. The cells were irregular polygonal, and the adjacent hepatocytes were closely connected to each other, forming island structure (Supplementary Fig 2. a, c). Meanwhile, Glycogen staining results showed that hepatocyte parenchyma was rich in glycogen which was stained with purple-red and distributed homogeneously and granularly (Supplementary Fig 2. b, d). There was no purple-red color was observed in the negative control group.

**Figure legends**

Figure 1 *In vitro* cultured chicken embryo primary intestine epithelial cells. A: Cell morphology after been cultivated for 48 hours (100×). B: Transmission electron microscope of chicken intestinal epithelial cells. (Abbreviations in Fig 1.B: D: Desmosome; Mit: Mitochondria; MV: Microvillus; Nu: Nucleus; RER: Rough Endoplasmic Reticulum; SV: Secretory Vesicle; TJ: Tight Junction). C: Immunofluorescence staining of cytokeratin 18 in chicken intestinal epithelium cells (IEC), chicken Esophageal Fibroblasts were used as the negative control (400×).

Figure 2 In vitro cultured chicken primary embryo hepatocytes by using periodic acid-Schiff (PAS). Glycogen can be oxidized to aldehydes by periodic acid, aldehydes react with SCHIFF reagents and produce purple-red compounds. Hepatocytes without oxidization by 0.5% periodic acid solution were used as the negative control. Negative control: a (200×), c (400×). PAS stained glycogen in primary hepatocytes: b (200×), d (400×).
